# Asian Pacific Islander Desi American college students and COVID-19-related racial discrimination: Mental health and the moderating role of ethnic identity

**DOI:** 10.1371/journal.pone.0309399

**Published:** 2024-10-16

**Authors:** Ronna Bañada, Hans Oh, Yuri Jang, Shinyi Wu, Joyce Javier, Jiaming Liang, Lawrence A. Palinkas

**Affiliations:** 1 Suzanne Dworak-Peck School of Social Work, University of Southern California, Los Angeles, California, United States of America; 2 Edward R. Roybal Institute on Aging, University of Southern California, Los Angeles, California, United States of America; 3 Department of Social Welfare, Ewha Womans University, Seoul, Republic of Korea; 4 Daniel J. Epstein Department of Industrial and Systems Engineering, Viterbi School of Engineering, University of Southern California, Los Angeles, California, United States of America; 5 Department of Health Systems Science, Kaiser Permanente Bernard J. Tyson School of Medicine, Pasadena, California, United States of America; 6 School of Public Health, University of Texas Health Science Center at Houston, Houston, Texas, United States of America; 7 Herbert Wertheim School of Public Health, University of California, San Diego, California, United States of America; National Institute of Preventive and Social Medicine, BANGLADESH

## Abstract

**Background:**

In 2020, the Coronavirus disease (COVID-19) triggered the latest wave of anti-Asian discrimination. During the first year of the pandemic, symptoms of depression and anxiety increased seven-fold within Asian Pacific Islander Desi American (APIDA) communities. Among this population, APIDA college students were at particularly high risk for mental health challenges due to COVID-19-related racial discrimination. This study examined the association between COVID-19-related racial discrimination and the mental health of APIDA college students, conceptualizing ethnic identity as a moderator in the association.

**Methods:**

Secondary analysis was conducted on data from 2,559 APIDA college students aged 18 to 29 who participated in the Fall and Winter/Spring Cohorts of the 2020–2021 Healthy Minds Study (HMS), a non-probability web-based survey administered to students in higher education in the United States. Descriptive statistics, comparative analysis (e.g., Chi-square and t-test), and multivariable linear regression were conducted using STATA 17.1 (StataCorp LLC, College Station, TX). Survey weights were applied in all analyses.

**Results:**

There were significant positive associations between COVID-19-related racial discrimination and symptoms of depression (*b* = 2.15, *p* < 0.001) and anxiety (*b* = 1.81, *p* < 0.001) among the overall sample. Furthermore, a greater sense of ethnic identity was associated with lower symptoms of depression (*b = -0*.*15*, *p*< 0.001) among the overall sample. Finally, ethnic identity buffered the association between COVID-19-related racial discrimination and symptoms of anxiety among East Asian students and symptoms of both depression and anxiety among Native Hawaiian and Pacific Islander students. In contrast, ethnic identity intensified the association between COVID-19-related racial discrimination and symptoms of depression among Filipino students.

**Conclusions:**

The research found that COVID-19-related racial discrimination was associated with increased symptoms of depression and anxiety among the full sample of APIDA college students during the first year of the pandemic. Additionally, higher levels of ethnic identity were associated with decreased depression among the entire group. The striking results on the moderating role of ethnic identity among subgroups call for further research on the ethnic identity development of APIDA college students, to help mitigate the effects of racial discrimination within a variety of systemic, complex, and dynamic sociocultural contexts.

## Introduction

Asian Pacific Islander Desi American (APIDA) communities are the fastest growing racial/ethnic groups in the United States, with a total of 20.6 million individuals making up 6.2% of the US population [1, 2]. In 2022, The U.S. Census Bureau published data from the 2016–2020 American Community Survey noting that there were 25 different groups that fall under the umbrella of APIDA, including Chinese, Indian, Filipino, Vietnamese, Korean, Native Hawaiian, Samoan, and Chamorro [2]. Serious psychological distress is also prevalent among these communities, such that over 2.8 million APIDA were estimated to have a mental health problem in 2019 [3–7]. Recent data from the 2015–2019 California Health Interview Survey (CHIS) showed that about 68% of all APIDA adults in California with moderate to serious psychological distress experienced unmet need for mental health care ranging from 45% of Japanese adults to 78% of Vietnamese adults [8]. Additionally, Filipinos are almost 1.5 times as likely to have serious psychological distress compared to all other Asian adults [8].

The upward trend of mental health challenges continued during the first year of the COVID-19 pandemic when APIDA individuals reported experiencing symptoms of depression and anxiety that were seven times higher compared to those reported in 2019 [9]. The introduction and spread of the coronavirus disease triggered harmful rhetoric, with prominent figures in society blaming the pandemic on people of Chinese origin and other diverse APIDA groups [9–11]. The surge in anti-Asian racism and discrimination during this time included verbal harassment and physical assaults [12, 13]. According to the Asian Pacific Policy and Planning Council (A3PCON), more than 40% of the reports were from Chinese individuals and other East Asian ethnic groups [9]. However, other APIDA communities were also targeted, based on their phenotypic characteristics, regardless of their ethnicity or nationality [9]. APIDAs have faced violence, verbal attacks, exclusion, disenfranchisement, microaggressions, and stereotypes across generations, and this historical and multigenerational transmission of trauma may also add to the distress that some communities may feel in the current sociopolitical climate [6, 14–16]. Because APIDA communities are routinely treated as a homogenous group, there is limited knowledge regarding the differences that can exist among these various communities and the experiences and identities that they hold [4].

Among APIDA populations, young adults are equally, if not more, vulnerable to developing mental health problems than their counterparts belonging to other racial/ethnic groups [4]. A particularly high-risk group is APIDA college students, 1.39 million of whom were enrolled in college in 2020, which was an 8.5% increase from the previous decade [17]. Literature suggests that the transition to post-secondary education is associated with increased stress which is related to societal and family expectations, academic demands, and interpersonal relationships [18]. Furthermore, symptoms of depression and anxiety have nearly doubled in the past decade in college populations [19]. Zhou and colleagues’ analysis of the Fall 2019 and Fall 2020 administrations of the Healthy Minds Study (HMS) (which represents the largest nationally recognized data of APIDA college student mental health symptoms and experiences of racial discrimination directly related to COVID-19) found that APIDA non-international students reported a 9% increase in severe depression and a 23% increase in severe anxiety, while APIDA international students reported a 16% increase in severe depression and a 12% increase in severe anxiety [20].

The transition to post-secondary education can also come with additional stressors, such as experiences of perceived discrimination. APIDA college students continue to experience increases in mental health needs because of stressors related to the COVID-19 pandemic, with Zhou et al. finding that around one in four APIDA college students reported experiencing pandemic-related racial discrimination or hostility [20]. Among APIDA young adults and college students, studies have shown that perceived discrimination was associated with mental health outcomes such as depression and anxiety [15, 21, 22]. However, it is unknown whether different subgroups of college-aged APIDAs were at risk for mental health problems specifically due to COVID-19-related racial discrimination, as other studies have examined experiences of discrimination and mental health outcomes before the pandemic, had smaller sample sizes, focused on APIDAs over the age of 30, analyzed data on APIDA participants as one combined group, or found that the relationship between pandemic-related racial discrimination and mental health outcomes did not vary among APIDA subgroups [9, 11, 20, 23, 24].

Guided by social identity theory and the protective model of resiliency, this study focused specifically on the association of COVID-19-related racial discrimination on the mental health of APIDA college students and the moderating role of ethnic identity in this relationship [25, 26]. Social identity theory posits that people naturally seek to have a positive self-concept and are also motivated to maintain this concept or image of themselves with their ethnocultural group [25]. When someone experiences racial discrimination (e.g., being rejected from the majority group) a person may look for belongingness and acceptance in their ethnocultural communities [25, 26]. According to Phinney, ethnic identity is a process in which people explore and define the meaningfulness of ethnicity in their lives [27]. Ethnic identity has been examined specifically regarding its role in psychological and developmental outcomes, for example, a longitudinal study on the mental health of Filipino and Korean adolescents (n = 781) showed that a greater sense of ethnic identity reduced symptoms of depression [4]. Similarly, another study found that higher ethnic identity was positively correlated with psychological well-being [28]. Along these lines, the risk-protective model of resiliency posits that positive contextual, social, and individual variables, also called *promotive resources*, can protect against the negative effects of risks and act as modifiers in the relationship between risks and deleterious outcomes [26]. These paradigms suggest that ethnic identity may serve as an important protective factor, or social and psychological resource, that helps minoritized populations like APIDAs respond with resilience when experiencing racial discrimination [28]. Therefore, ethnic identity may influence an APIDA college student’s perception of race-related stressors, such as racial discrimination, as well as the extent to which they experience those stressors as mentally or emotionally distressing [29].

Promotive resources such as ethnic identity may provide a sense of belonging and solidarity with a person’s ethnic community and increase their positive view of their ethnic identity in response to experiences of racial discrimination [26, 28]. Earlier studies have identified ethnic identity as an important protective factor against racism and discrimination for APIDAs [28, 30]. For example, a survey of Filipino Americans (N = 2,109) in San Francisco, CA, found that ethnic identity served as a protective factor, buffering the relationship between experiences of lifetime racial discrimination and mental health and significantly reducing symptoms of depression [30]. Similarly, a study on COVID-19-related racial discrimination experienced by Chinese immigrants and Chinese Americans (N = 187) ages 16 to 63 found that high levels of ethnic identity buffered the relationship between pandemic-related discrimination and depression [28]. Woo and colleagues found that while ethnic identity differentially moderated the relationship between racial discrimination and mental health among various racial/ethnic groups, a moderate level of ethnic identity buffered the impact of discrimination on the mental health of Asian participants in their study [29].

Conversely, ethnic identity may exacerbate the “psychological burden” of discrimination for those individuals who put stronger values on their ethnic [identity and] background [29]. The more prominent a person holds their ethnic identity, the more importance and purpose they may attach to that identity, which may then have more influence on their mental health [29]. For example, a quasi-experimental study by Yoo and colleagues found that Asian American individuals who had strong ethnic identities, experienced decreased psychological well-being when they envisioned experiencing multiple incidents of racial discrimination [31]. Evidence from another study showed that having a strong ethnic identity intensified this association between perceived discrimination and anxiety among East Asian and Southeast Asian adults [32]. To add further variability to the results of previous studies, Lee found that ethnic identity did not moderate the association between discrimination and well-being in a sample of APIDA college students [28].

Extant research on the mental health outcomes of APIDA college students has often categorized them as one monolithic group, which may not reflect the range of mental health challenges among diverse groups of APIDAs [11, 29, 33]. The increasing numbers of APIDA students in higher education combined with the surge in anti-Asian discrimination, exacerbated by the global pandemic, calls for more research on the mental health experiences of this population. To our knowledge, no other studies have empirically explored whether a sense of ethnic identity moderated the association between COVID-19-related racial discrimination and mental health among APIDA students in higher education. To expand on existing research on the effects of COVID-19-related racial discrimination among APIDA college students, this study examined the following hypotheses: (1) COVID-19-related racial discrimination would be positively associated with symptoms of depression and anxiety, (2) A greater sense of ethnic identity would be associated with lower symptoms of depression and anxiety, (3) Ethnic identity would moderate the association between COVID-19-related racial discrimination and symptoms of depression and anxiety. These associations were also explored across ethnic subgroups.

## Methods

### Data and participants

Secondary data for this study were drawn from the Fall and Winter/Spring cohorts of the 2020–2021 Healthy Minds Study (HMS), a non-probability web-based survey administered to students enrolled in higher education in the United States [34]. The HMS data were collected under the approval of Advarra and the Institutional Review Boards at all participating colleges and universities (IRB number: Pro00028565). The studies involving human participants were reviewed and approved by University of Michigan. The student participants provided their written informed consent to participate in this study. The secondary analysis presented in this study was deemed exempt under the approval of USC (UP-22-00068). The HMS survey is administered annually to a cross-section of schools, with a different set of schools every year, including community colleges, four-year colleges, and professional schools. The specific regions of data collection were not publicly available. The HMS survey uses several validated measures to provide information about the prevalence of mental health outcomes, knowledge, attitudes about mental health, and service utilization. The survey was administered to 34,168 students attending 37 institutions of higher learning between September through December 2020 and then administered again to 103,748 students at 103 institutions between January through June 2021. The response rate was 14%. We restricted the sample by focusing on students identifying as APIDA aged 18–29. We used complete-case analysis, resulting in a final analytic sample (n = 2,559).

### Inclusion and exclusion criteria

The inclusion criteria used in this research were as follows: i) English-speaking, ii) currently enrolled students at higher education institutions in the U.S., ii) over 18 years old. The exclusion criteria used in this study were as follows: i) non-English-speaking, ii) students under the age of 17.

### Measures

#### Demographic variables

Ethnicity (0 = East Asian (e.g., Chinese, Japanese, Korean, Taiwanese), 1 = Southeast Asian (e.g., Cambodian, Vietnamese, Hmong), 2 = Desi/South Asian (e.g., Indian, Pakistani, Nepalese, Sri Lankan), 3 = Filipina/x/o, 4 = Native Hawaiian or Pacific Islander, 5 = Multi-ethnic. Age (18–29) was treated as a continuous variable. Gender identity (0 = man, 1 = woman), sexual orientation (0 = straight, 1 = lesbian, gay, bisexual, queer, or other), international student (0 = no, 1 = yes), and socioeconomic status (0 = parental education college and above, 1 = parental education less than college).

#### Predictor

*COVID-19-related racial discrimination*. COVID-19-related racial discrimination was measured using the item: “As a result of the COVID-19 pandemic, have you experienced any discriminatory or hostile behavior due to your race/ethnicity (or what someone thought was your race/ethnicity)?” Responses were coded into a binary format (0 = no, 1 = yes).

#### Outcomes

*Mental health*. As indicators of mental health, we used symptoms of depression and anxiety, the two most prevalent mental health problems among college students [34]. Depressive symptoms were measured with the Patient Health Questionnaire (PHQ-9) [35]. For this study, the PHQ-9 was treated as a severity measure and operationalized as a continuous variable [35].

Each item was measured on a 4-point Likert scale from 0 (Not at all) to 3 (Nearly every day). Total scores range from 0 to 27, and the scale presents a high level of internal consistency (α = 0.89). The PHQ-9 has been validated with good reliability and validity among a similar population of Asian American college students [15]. Anxiety was measured with the 7-item anxiety scale (GAD-7), a validated tool for screening Generalized Anxiety Disorder and assessing its severity [36]. For this study, the GAD-7 was treated as a severity measure and operationalized as a continuous variable [36]. Each item was measured on a 4-point Likert scale of 0 (Not at all) to 3 (Nearly every day). Total scores range from 0 to 21, and the scale presents a high level of internal consistency (α = 0.92). The GAD-7 has also been validated with good reliability and validity among another study on Asian American college students [15].

#### Moderator

*Ethnic identity*. Ethnic identity was measured using a six-item version of the Multi-group Ethnic Identity Measure (MEIM), which has been psychometrically tested with diverse populations, including different age groups, ethnic backgrounds, and locations [21, 31, 37, 38]. In this study ethnic identity was conceptualized as a continuous variable, where low scores indicate lower interest, awareness, or clarity regarding one’s ethnicity, while higher scores are indicative of increased efforts to learn more about one’s background and greater commitment to understand the role of ethnicity for one’s own identity [37]. The measure includes: “Being a member of my racial/ethnic group is an important reflection of who I am,” “I have a strong sense of belonging with other people in my racial/ethnic group,” and “I have a strong attachment to other people in my racial/ethnic group.” Each item was measured on a 5-point Likert scale from 1(Strongly disagree) to 5 (Strongly agree). Total scores range from 6 to 30 and the measure presents a high level of internal consistency (α = 0.87).

### Analytic strategies

Secondary analyses were conducted using STATA 17.1 [39]. Survey weights were used in all analyses to account for non-response based on the known student populations at each school. Descriptive statistics (frequencies, percentages) of the overall sample and each of the subgroups were examined. Subgroup differences were evaluated using Chi-square (for the categorical variables) and t-test (for the continuous variables) analyses and the East Asian subgroup was used as a reference group because they are the largest and most-studied ethnic group of Asian Americans [40]. The PHQ-9 and GAD-7 scores were not normally distributed, therefore, we conducted a log transformation of the dependent variables to reduce the skewness and kurtosis within the normal range. We calculated bivariate correlations among the study variables to understand their underlying associations and ensure the absence of multicollinearity. Guided by a theory-driven model we used multivariable linear regression models of depressive symptoms and anxiety to test direct and moderating effects of COVID-19-related racial discrimination, ethnic identity, and ethnicity. All analyses were conducted with adjustment for sociodemographic variables (age, gender, sexual orientation, citizenship, socioeconomic status). In each model of mental health outcomes, we first tested the direct effects of discrimination (Model 1) and ethnic identity (Model 2), as well as their interaction (Model 3). The subsequent models examined potential ethnic group variations in the effects of discrimination (Model 4) and ethnic identity (Model 5). The final model (Model 6) with a three-way interaction examined how the moderating role of ethnic identity in the association between discrimination and mental health would vary by ethnicity. When an interaction term was found to be significant, the overall sample was divided into subgroups based on the moderating variable (i.e., ethnic identity, ethnicity), and subgroup differences in the effect of a predictor variable on mental health outcome were examined.

## Results

### Description of the sample

Table 1 showed the descriptive characteristics of the study sample. The largest ethnic subgroup was East Asians (n = 1,072, 41.9%), and the smallest ethnic subgroup was multi-ethnic APIDAs (n = 127, 5.0%). Of all 2,559 participants, the mean age was 21.4 (SD = 3.0) with a range of 18–29, and 69.1% identified as women and 30.9% identified as men. More than 22% of the overall sample identified as lesbian, gay, bisexual, queer, or other, with a range from 15.1% among Desi-South Asian students, to 32.3% among Filipino students. Furthermore, 16% of the overall sample were international students, with a range from 2.3% among Filipino, Native Hawaiian, and Pacific Islander students, to 20.5% among Desi/South Asian students. Among the overall sample, almost 27% of students had parents who had educational levels below college, with a range from 18.1% among Filipino students to 47.8% among Southeast Asian students. Nearly a quarter (24%) of the overall sample experienced COVID-19-related racial discrimination, with a range from 5.2% among Desi/South Asian Americans to 37% among multi-ethnic APIDA students. The overall sample of APIDA students scored a mean of 20.0 (SD = 5.3) on the Multi-group Ethnic Identity Measure (MEIM), with a range from 19.5 (SD = 5.6) among Native Hawaiian and Pacific Islander students, to 20.4 (SD = 5.1) among Filipino students. For symptoms of depression, students had a mean score of 9.4 (SD = 6.7) on the PHQ-9, with a range from 8.7 (SD = 6.3) among East Asian students, to 11.3 (SD = 7.30) among Native Hawaiians or Pacific Islander students. For anxiety symptoms, students had a mean score of 7.7 (SD = 5.9) on the GAD-7, with a range from 7.0 (SD = 5.6) among East Asian students to 9.2 (SD = 6.0) among Native Hawaiian or Pacific Islander students. The highest correlation was observed between sexual orientation and depression (Spearman’s rho = 0.27, p < .001); those who identified as LGBQ+ were more likely to also experience higher symptoms of depression. We also conducted additional analyses to determine that multicollinearity was not a concern, with VIF values that ranged from 1.01 to 1.19 among the study variables.

**Table 1:** Descriptive characteristics of the sample.

	Mean ± SD or %						
	Overall sample (*N =* 2,559)	East Asian (*n* = 1,072)	Southeast Asian (*n* = 359)	Desi/South Asian (*n* = 653)	Filipino (*n* = 219)	Native Hawaiian/ Pacific Islander (*n* = 129)	Multi-ethnic (*n* = 127)
Age (years)	21.4 ± 3.0	21.6 ± 3.0	20.9 ± 2.6***	21.5 ± 3.1	20.9 ± 2.7***	21.1 ± 2.8	21.0 ± 2.7**
Gender							
Woman	69.1	67.7	71.8*	67.1	73.6*	70.2	74.2
Sexual orientation							
LGBQ+	22.2	22.7	23.7	15.1***	32.3**	29.5	24.6
International student	16.4	19.6	16.2***	20.5	2.3***	2.3***	7.1***
SES (Parental education < college)	26.5	24.9	47.8***	20.7*	18.1	25.0	27.8
Covid-related racial discrimination	24.0	32.7	33.4	5.2***	21.9**	11.6***	37.0
Ethnic identity (MEIM-6)	20.0 ± 5.3	19.9 ± 5.3	20.3 ± 5.2	19.7 ± 5.5*	20.4 ± 5.1	19.5 ± 5.6	19.9 ± 5.0
Depression (PHQ-9)	9.4 ± 6.7	8.7 ± 6.3	9.9 ± 6.5***	9.4 ± 7.0**	10.4 ± 6.6***	11.3 ± 7.3***	10.3 ± 7.2**
Anxiety (GAD-7)	7.7 ± 5.9	7.0 ± 5.6	7.9 ± 5.5***	7.9 ± 6.2***	8.5 ± 5.9***	9.2 ± 6.0***	8.8 ± 6.5***

Note. χ^2^ and t-test analyses were conducted by comparing each ethnic group with East Asian;

* *p* < .05.

** *p* < .01.

*** *p* < .001

### Multivariable linear regression analyses on depressive symptoms

Table 2 showed the regression analyses on depressive symptoms. For each outcome, the same set of predictive models (models 1 to 6) was tested for the proposed hypotheses. In the analyses on depressive symptoms, Model 1 showed that COVID-19-related racial discrimination was positively related to symptoms of depression (*b* = 2.15, *p* < 0.001). In Model 2, ethnic identity was negatively associated with depressive symptoms (*b* = -0.15, *p* < 0.001). Model 3 showed that, among the overall sample, ethnic identity did not moderate the association between COVID-19-related racial discrimination and symptoms of depression. Similarly, in Model 4, ethnicity did not moderate the association between COVID-19-related racial discrimination and depression among the overall sample. In Model 5, compared to East Asian students, higher ethnic identity among Desi/South Asian students was associated with lower symptoms of depression. Model 6 showed there were significant differences across ethnic subgroups when analyzing three-way interactions among COVID-19-related racial discrimination, ethnic identity, and ethnicity for symptoms of depression.

**Table 2:** Regression models of COVID-19-related racial discrimination and depression symptoms among the full sample.

	B (SE)					
	Model 1	Model 2	Model 3	Model 4	Model 5	Model 6
Discrimination	2.15*** (0.31)		2.21*** (0.43)	2.16*** (0.53)	2.26*** (0.32)	2.39*** (0.65)
Ethnic identity		-0.15*** (0.03)	-0.17*** (0.03)	-0.16*** (0.03)	-0.10* (0.04)	-0.08 (0.05)
Disc x EID			0.02 (0.08)			
Ethnic subgroup						
East Asian	[ref]	[ref]	[ref]	[ref]	[ref]	[ref]
Southeast Asian	0.85 (0.56)	0.90 (0.53)	0.91 (0.55)	0.80 (0.79)	1.04 (0.65)	1.04 (0.90)
Desi/South Asian	1.83** (0.53)	1.29* (0.49)	1.84*** (0.49)	1.89** (0.53)	2.25*** (0.49)	2.36*** (0.53)
Filipino	0.93 (0.81)	0.80 (0.80)	1.00 (0.78)	0.97 (0.89)	1.18 (0.95)	1.46 (1.08)
NHPI	2.11* (0.92)	1.69 (0.96)	2.13* (0.96)	2.18* (1.00)	2.44* (1.07)	2.43* (1.12)
Multi-ethnic	0.84 (0.90)	1.02 (0.90)	0.79 (0.90)	-0.28 (1.17)	0.90 (1.38)	-0.31 (1.54)
Disc x Ethnicity						
Disc x EA				[ref]		
Disc x SEA				0.37 (1.48)		
Disc x DSA				-1.71 (1.09)		
Disc x FI				0.04 (1.42)		
Disc x NHPI				-0.92 (1.95)		
Disc x ME				2.68 (1.58)		
EID x Ethnicity						
EID x EA					[ref]	
EID x SEA					-0.04 (0.08)	
EID x DSA					-0.15* (0.06)	
EID x FI					-0.06 (0.12)	
EID x NHPI					-0.11 (0.16)	
EID x ME					-0.04 (0.21)	
Disc x EID x Ethnicity						
Disc x EID x EA						[ref]
Disc x EID x SEA						0.15 (0.19)
Disc x EID x DSA						0.10 (0.22)
Disc x EID x FI						0.56** (0.17)
Disc x EID x NHPI						-0.64 (0.35)
Disc x EID x ME						-0.17 (0.33)
Overall R^2^	0.11***	0.11***	0.13***	0.13***	0.13***	0.13***

Notes:

a. Disc = Covid-19-related racial discrimination; EID = Ethnic identity; EA = East Asian; SEA = Southeast Asian; DSA = Desi/South Asian; FI = Filipino; NHPI = Native Hawaiian/Pacific Islander; ME = Multi-ethnic.

b. All models reflect the full sample of APIDA students and include the following covariates: age, gender, international student status, parental education.

c. Model 1 with direct effect of discrimination on depression including covariates; without interaction terms.

d. Model 2 with direct effect of ethnic identity on depression including covariates; without interaction terms.

e. Model 3 (with two-way interaction between COVID-19-related racial discrimination and ethnic identity) but no significant interaction was found.

f. Model 4 (with two-way interaction between COVID-19-related racial discrimination and ethnicity) but no significant interaction was found.

g. Model 5 (with two-way interaction between ethnic identity and ethnicity) found that compared to East Asian students, higher ethnic identity among Desi/South Asian students was associated with lower symptoms of depression.

h. Model 6 (with three-way interaction among COVID-19-related racial discrimination, ethnic identity, and ethnicity) found that having higher ethnic identity among Filipino students, compared to East Asian students, intensifies the association between COVID-19-related racial discrimination and symptoms of depression.

### Multivariable linear regression analyses on symptoms of anxiety

Table 3 showed regression analyses on anxiety symptoms. According to Model 1, COVID-19-related racial discrimination was positively related to symptoms of anxiety (b = 1.81, *p* < 0.001). In Model 2, ethnic identity was not significantly associated with anxiety symptoms (*b* = -0.06, *p* > 0.05). For Model 3, ethnic identity did not moderate the association between COVID-19-related racial discrimination and symptoms of anxiety among the overall sample. In Model 4, ethnicity significantly moderated the association between COVID-19-related racial discrimination and anxiety, specifically showing that compared to East Asians, being Desi/South Asian and experiencing COVID-19-related racial discrimination was associated with decreased symptoms of anxiety. In Model 5, there were no significant interactions among ethnic identity and ethnicity when looking at anxiety symptoms. Model 6 showed there were significant differences across ethnic subgroups when analyzing three-way interactions among COVID-19-related racial discrimination, ethnic identity, and ethnicity for symptoms of anxiety. This led us to further investigate each ethnic subgroup separately to determine whether ethnic identity moderated the relationship between COVID-19-related racial discrimination and symptoms of depression and anxiety.

**Table 3:** Regression models of COVID-19-related racial discrimination and anxiety symptoms among the full sample.

	B (SE)					
	Model 1	Model 2	Model 3	Model 4	Model 5	Model 6
Discrimination	1.81*** (0.29)		2.22*** (0.37)	2.19*** (0.44)	1.86*** (0.28)	2.92*** (0.57)
Ethnic identity		-0.06 (0.03)	-0.05 (0.03)	-0.07* (0.03)	-0.02 (0.04)	0.03 (0.04)
Disc x EID			-0.10 (0.07)			
Ethnic subgroup						
East Asian	[ref]	[ref]	[ref]	[ref]	[ref]	[ref]
Southeast Asian	0.68 (0.50)	0.70 (0.48)	0.71 (0.50)	0.83 (0.78)	1.06 (0.61)	1.32 (0.82)
Desi/South Asian	2.07*** (0.44)	1.63*** (0.42)	2.06*** (0.43)	2.29*** (0.43)	2.38*** (0.49)	2.76*** (0.49)
Filipino	0.96 (0.63)	0.81 (0.64)	0.99 (0.62)	1.30 (0.65)	0.74 (0.63)	1.23 (0.67)
NHPI	1.77** (0.63)	1.42* (0.66)	1.79** (0.64)	1.93** (0.67)	1.98* (0.80)	2.16* (0.82)
Multi-ethnic	1.37 (0.87)	1.55 (0.87)	1.34 (0.85)	0.59 (1.06)	1.55 (1.25)	0.54 (1.24)
Disc x Ethnicity						
Disc x EA				[ref]		
Disc x SEA				-0.40 (1.45)		
Disc x DSA				-2.72* (1.07)		
Disc x FI				-1.45 (1.11)		
Disc x NHPI				-1.12 (1.89)		
Disc x ME				1.74 (1.31)		
EID x Ethnicity						
EID x EA					[ref]	
EID x SEA					-0.11 (0.08)	
EID x DSA					-0.11 (0.07)	
EID x FI					0.07 (0.10)	
EID x NHPI					-0.07 (0.15)	
EID x ME					-0.07 (0.19)	
Disc x EID x Ethnicity						
Disc x EID x EA						[ref]
Disc x EID x SEA						0.25 (0.23)
Disc x EID x DSA						0.46* (0.23)
Disc x EID x FI						0.36* (0.18)
Disc x EID x NHPI						-0.43 (0.29)
Disc x EID x ME						-0.23 (0.26)
Overall R^2^	0.12***	0.10***	0.12***	0.12***	0.12***	0.13***

Notes:

a. Disc = Covid-19-related racial discrimination; EID = Ethnic identity; EA = East Asian; SEA = Southeast Asian; DSA = Desi/South Asian; FI = Filipino; NHPI = Native Hawaiian/Pacific Islander; ME = Multi-ethnic.

b. All models reflect the full sample of APIDA students and include the following covariates: age, gender, international student status, parental education.

c. Model 1 with direct effect of discrimination on depression including covariates; without interaction terms.

d. Model 2 with direct effect of ethnic identity on depression including covariates; without interaction terms.

e. Model 3 (with two-way interaction between COVID-19-related racial discrimination and ethnic identity) but no significant interaction was found.

f. Model 4 (with two-way interaction between COVID-19-related racial discrimination and ethnicity) found that Desi/South Asian students who experienced COVID-19-related racial discrimination, compared to East Asian students, have decreased symptoms of anxiety.

g. Model 5 (with two-way interaction between ethnic identity and ethnicity) but no significant interactions were found.

h. Model 6 (with three-way interaction among COVID-19-related racial discrimination, ethnic identity, and ethnicity) found that having higher ethnic identity among Desi/South Asian and Filipino students, compared to East Asians, intensifies the association between COVID-19-related racial discrimination and symptoms of anxiety.

Among East Asian students, increased ethnic identity buffered the relationship between COVID-19-related racial discrimination and depressive symptoms. Among Native Hawaiian and Pacific Islander students, increased ethnic identity buffered the relationship between COVID-19-related racial discrimination and symptoms of depression and anxiety. Conversely, among Filipino students, ethnic identity intensified the relationship between COVID-19-related racial discrimination and symptoms of depression.

**Fig 1:**
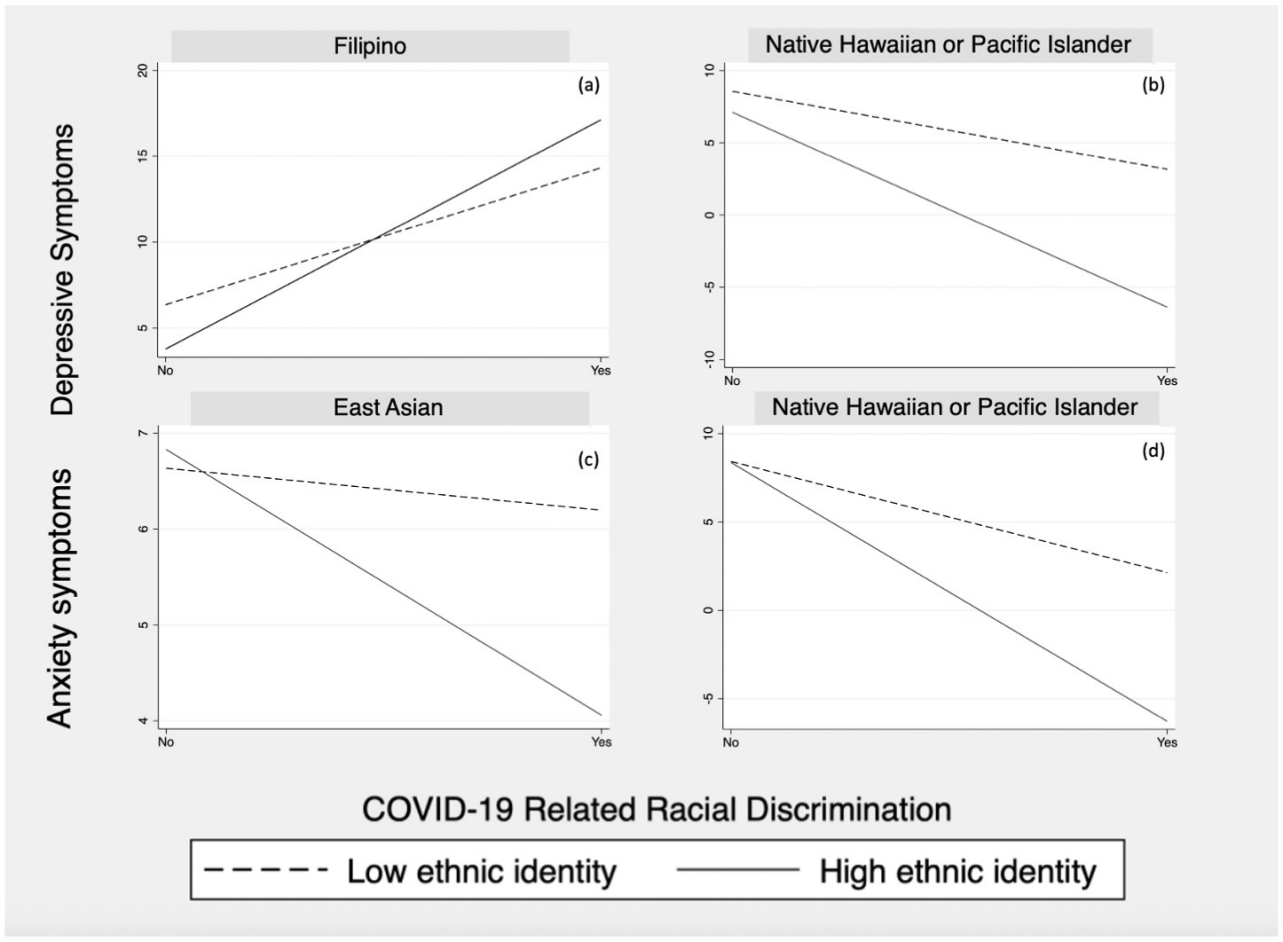
Ethnic identity as a moderator among APIDA students. Notes: a. Fig 1(a) shows that ethnic identity moderates the association between COVID-19-related racial discrimination and depressive symptoms among Filipino students (Low ethnic identity: B = -4.93, p <0.05; High ethnic identity: B = 3.93, p > 0.05). b. Fig 1(b) shows that ethnic identity moderates the association between COVID-19-related racial discrimination and depressive symptoms among Native Hawaiian and Pacific Islander students (Low ethnic identity: B = 12.18, *p* <0.01). c. Fig 1(c) shows that ethnic identity moderates the association between COVID-19-related racial discrimination and anxiety symptoms among East Asian students (Low ethnic identity: B = 4.78, *p* <0.01; High ethnic identity: B = 3.01, *p <* 0.05). d. Fig 1(d) shows that ethnic identity moderates the association of COVID-19-related racial discrimination and anxiety symptoms among Native Hawaiian and Pacific Islander students (Low ethnic identity: B = 8.64, *p* <0.001).

Fig 1 showed the significant interaction effects of ethnic identity on the relationship between COVID-19-related racial discrimination and mental health outcomes among specific ethnic subgroups, where the relationship between COVID-19-related racial discrimination (X-axis) and symptoms of depression and anxiety (Y-axis) was plotted for low and high ethnic identity. As can be seen in the figures, the association of COVID-19-related racial discrimination (i.e., the differences between the dashed and solid line slopes) changed as one’s ethnic identity increased from “low” to “high.

## Discussion

This study provided unique findings around the associations between COVID-19-related racial discrimination, ethnic identity, and mental health among APIDA students in higher education during the first year of the pandemic. It also provided important insights into the role of ethnic identity by analyzing the overall sample of APIDA college students as well as specific subgroups within this population. Our first hypothesis was supported and showed that COVID-19-related racial discrimination was significantly associated with increased symptoms of depression and anxiety among the full sample. Our second hypothesis was partially supported and showed that higher ethnic identity was associated with decreased symptoms of depression among the full sample. Contrary to our third hypothesis, ethnic identity did not moderate the association between COVID-19-related racial discrimination and mental health outcomes among the full sample. However, after subgroup analysis, our study revealed that ethnic identity buffered the relationship between COVID-19-related racial discrimination and mental health symptoms among East Asian (depression), Native Hawaiian, and Pacific Islander (depression and anxiety) students. In contrast, the opposite occurred for Filipino students–higher ethnic identity intensified the relationship between COVID-19-related racial discrimination and symptoms of depression.

As hypothesized, the finding that racial discrimination was associated with both mental health outcomes is consistent with extant research which showed that APIDAs who experienced racial discrimination had greater symptoms of depression and anxiety [15, 21, 22]. Additionally, the finding that stronger ethnic identity reduced symptoms of depression among the entire sample of APIDA students aligns with social identity theory. This supports previous empirical studies highlighting the positive relationship between ethnic identity and decreased mental health symptoms in APIDA populations [4].

We contributed to the literature with our unique analysis of various ethnic subgroups of APIDA college students which provided expanded insights into how different groups have been impacted by COVID-19-related racial discrimination [20]. Partially supporting the risk-protective model of resiliency, this study underscored the complex role of ethnic identity as a moderating factor. Stronger ethnic identity functioned as a protective factor against negative mental health for East Asian, Native Hawaiian, and Pacific Islander students who experienced COVID-19-related racial discrimination. However, it exacerbated the association between COVID-19-related racial discrimination and symptoms of depression among Filipino students. These opposing results reflect the mixed findings of prior studies on ethnic identity as a moderating factor [28, 31, 32]. The current study found that ethnic identity helped to reduce symptoms of anxiety among East Asian students who experienced COVID-19-related racial discrimination. These findings were different from a previous study on East Asian adults that showed stronger ethnic identity exacerbated the relationship between discrimination and anxiety [32]. To support the results in the study conducted by Woo and colleagues, this study showed that the moderating effect of ethnic identity on the association between COVID-19-related racial discrimination and mental health outcomes is not linear but dependent on the level of ethnic identity [29]. The findings from this current study diverge from an earlier one where ethnic identity was found not to moderate the effects of discrimination on well-being [28]. This current study had a larger sample size and, therefore, was able to analyze APIDA subgroups to find different effects.

Notably, the disaggregation of data on Native Hawaiian and Pacific Islander students in this current study provided insights into the role of ethnic identity as a promotive resource and protective factor for these communities. The results underscore the importance of examining specific APIDA ethnic groups to provide valuable insights on unique ways to support the mental health needs in these communities. For example, the study results provided a counternarrative to other empirical studies on the buffering role of ethnic identity on mental health among Filipino American adults who experienced racial discrimination [30]. The current study focused on a younger age group, specifically Filipino college students who experienced COVID-19-related racial discrimination, during a time of heightened sociopolitical strife in the United States [30]. The differing results also highlight how various APIDA ethnic groups possess unique historical and sociopolitical positions in U.S. society. These positions influence their experiences and understanding of group membership, as well as their diverse beliefs, perspectives, and responses to racial discrimination [10, 16, 27, 41].

### Limitations

This present study should be interpreted in light of some limitations. Due to its cross-sectional and non-probability design, causal inferences cannot be drawn, and the findings may not generalize to the larger population of APIDAs who reside in various regions of the country. Another limitation is the potential for nonresponse bias, although a 14% response rate is not uncommon for online surveys [43]. Also, COVID-19-related racial discrimination was measured using a single ‘yes’ or ‘no’ question, and future research should use more nuanced discrimination measures. We were unable to account for all confounders related to COVID-19-related racial discrimination, depression, and anxiety, and therefore our findings may have been subject to omitted variable biases. However, the HMS is one of the few datasets that has captured pandemic-related racial discrimination, and, despite its limitations, the data are novel and provide important insights into the lives of young APIDA college students in the first year of the pandemic. Further, ethnic identity is complex. We also used a composite score for ethnic identity that was divided into low and high categories. However, there is a lot of variability in how researchers have measured ethnic identity [28, 29, 31]. Without a comprehensive list of confounders, it is possible that the moderating effect of ethnic identity may have also intervened on other covariates. Future research may benefit from the inclusion of scales that measure the multidimensional constructs of ethnic and racial identities, separately, to better capture the complex experiences of diverse APIDA college students.

## Conclusion

The present study provides evidence of the complex mental health outcomes associated with COVID-19-related racial discrimination and further illuminates the role that ethnic identity plays among diverse groups of APIDA college students. We contributed to the literature with our analysis of ethnic subgroups whose lived experiences warrant deeper attention in social science and mental health research. Following social identity theory, researchers need to further examine the various ways that APIDA college students explore and understand their ethnic identity to help mitigate the effects of racial discrimination within a variety of systemic and complex sociocultural contexts. Future studies would also benefit from utilizing group-specific measures, such as the Colonial Mentality Scale, which may contribute to or interact with racial and/or ethnic identification to influence levels of psychological well-being or distress when experiencing racial discrimination [42, 43]. This approach would be beneficial to diverse groups of APIDA college students, whose sociocultural influences and experiences have led to diversity in racial and ethnic identification [41, 44]. Our study calls for more research to shape policy development, educational institutions, and healthcare delivery systems to collaborate with and support the efforts of community programs and initiatives focusing on APIDA mental health.
